# Oviposition behavior of wild yellow fever vector mosquitoes (Diptera: Culicidae) in an Atlantic Forest fragment, Rio de Janeiro state, Brazil

**DOI:** 10.1038/s41598-021-85752-y

**Published:** 2021-03-16

**Authors:** Shayenne Olsson Freitas Silva, Cecilia Ferreira de Mello, Ronaldo Figueiró, Tatiana Docile, Michele Serdeiro, Fabiana Fagundes Fumian, Jeronimo Alencar

**Affiliations:** 1 Diptera Laboratory, Oswaldo Cruz Institute (Fiocruz), Av. Brasil 4365, Rio de Janeiro, 21040-360 Brazil; 2 Postgraduate Program in Tropical Medicine, Oswaldo Cruz Institute (Fiocruz), Av. Brasil 4365, Rio de Janeiro, 21040-360 Brazil; 3 Postgraduate Program in Animal Biology, Institute of Biology, Federal Rural University of Rio de Janeiro, Seropédica, Rio de Janeiro, 23890-000 Brazil; 4 Foundation State University Center of the West Zone (UEZO), Av. Manuel Caldeira de Alvarenga, Rio de Janeiro, 120323070-200 Brazil; 5 Volta Redonda University Center (UniFOA), Av. Paulo Erlei de Abrantes, 1325, Volta Redonda, RJ 27240-560 Brazil; 6 Castelo Branco University (UCB), Av. Santa Cruz, 1631, Rio de Janeiro, 21710-250 Brazil; 7 Laboratory of Professional Education in Health Surveillance (LAVSA), Joaquim Venâncio Polytechnic School of Health, Oswaldo Cruz Foundation (FIOCRUZ), Avenida Brasil, Manguinhos, Rio de Janeiro, 436521040-900 Brazil; 8 Application Institute Fernando Rodrigues da Silveira (Cap-UERJ), State University of Rio de Janeiro (UERJ), Rio de Janeiro, Brazil

**Keywords:** Ecology, Zoology

## Abstract

Although there are many studies on the control of mosquito vectors of the yellow fever virus (YFV) in tropical forests, there are still few ecological studies regarding abiotic factors effect on these mosquitoes. Here we characterize these effects on oviposition behavior, abundance, and diversity of mosquito vectors of YFV. The study was conducted in Córrego da Luz Municipal Park, in Casimiro de Abreu, Rio de Janeiro state, Brazil, from July 2018 to December 2019. Ovitraps were placed at ground level and 3 m high. The data were tested for normality using the Shapiro–Wilk test, followed by an independent sample analysis, the Mann–Whitney test. The Shannon Diversity Index was used to evaluate the abundance of mosquitos' eggs collected at both ground level and 3 m high. We highlight the presence of *Haemagogus janthinomys* and *Hg. leucocelaenus*, primary YFV vectors in forest areas. The abundance of *Hg. leucocelaenus* (63%)*, Hg. janthinomys* (75%), and *Aedes terrens* (58%) was higher at the height of 3 m, while *Ae. albopictus* (52%) was higher at ground level. *Aedes albopictus* was positively correlated with temperature. Culicidae monitoring is essential for assessing the YFV transmission cycle in Atlantic forest fragments.

## Introduction

The interference of anthropic activities such as biome fragmentation, the introduction of exotic species, climate change, and environmental pollution in the ecosystem can significantly impact biodiversity, causing a decrease in species richness that leads to an imbalance in the natural cycles of an ecosystem^1,2^. The effects of these changes on climate variables lead to favorable conditions for mosquito proliferation^3,4^. There has therefore been an increasing expansion of mosquito-borne diseases associated with climate change and habitat destruction^5,6^.

Mosquitoes have attracted considerable attention due to their role in the transmission of several major pathogens, such as arboviruses (e.g., Zika, dengue, chikungunya, and yellow fever) and malaria. These illnesses are considered a serious worldwide public health problem, especially in tropical countries, like Brazil^7^.

Mosquitoes belonging to the genus *Haemagogus* and *Sabethes* are epidemiologically important as they act as biological vectors in the transmission of the wild yellow fever virus (YFV) in forest areas of the Americas^8^. *Haemagogus* species are diurnal mosquitoes most frequently found near the treetops in forest fragments. The height at which their acrodendrophilic activity occurs, also known as their vertical distribution, is quite wide and influences their oviposition behavior^9,10^.

According to Pessanha^11^, an increasing number of cases of zoonotic diseases have been noticed in transition areas over the last three decades as the process of deforestation has intensified.

The Brazilian coast from the state of Piauí to Rio Grande do Sul was considered a YFV-free area until 2016 and, consequently, was not subject to vaccination recommendations. However, a serious outbreak was observed in center-western Brazil between 2014 and 2015 that quickly spread through the most populous region of the Atlantic Forest, which had not seen circulation of the virus in almost 80 years^12^. The pathogen quickly spread through this heavily populated region, resulting in a severe outbreak from 2016 to 2018. In 2017, yellow fever cases were detected in Casimiro de Abreu, Rio de Janeiro state, resulting in one fatality. A comprehensive entomological survey by Abreu et al.^12^, conducted in 44 municipalities in five Brazilian states, analyzed the distribution, abundance, and natural infection by YFV of mosquitoes captured before and during the outbreak. The authors found that *Haemagogus janthinomys* Dyar, 1921 and *Hg. leucocelaenus* Dyar & Shannon, 1924 were the primary vectors due to their wide distribution, high abundance, and natural infection rates. The results of Abreu et al.^12^ highlight the important role of these species in the rapid spread and severity of this outbreak.

A clearer understanding of mosquito communities’ biodiversity in forest environments such as the Atlantic Forest, either during anthropic activities or periods of vegetation recovery, is crucial to understanding changes in behavior patterns among these wild mosquito populations.

Although the monitoring of mosquito vectors of YFV in tropical forests is the subject of a large body of literature^10,13^, there are still few ecological studies that focus on changes in the populations and communities of these vectors in fragmented forested environments, especially on how climatic factors affect the oviposition behavior of these mosquitoes.

In this study, we evaluate the influence of oviposition behavior and abiotic factors (temperature and rainfall) on the abundance and diversity of mosquito vectors of YFV from July 2018 to December 2019 in a remnant of Atlantic Forest of Córrego da Luz Municipal Park, Casimiro de Abreu city, Rio de Janeiro state, Brazil.

## Results

During the study period from July 2018 to December 2019, 6535 mosquito eggs were collected in the Córrego da Luz Municipal Park area. Of this total, 1449 eggs reached the adult stage, and four species were identified: *Hg. leucocelaenus*, *Hg. janthinomys*, *Ae. albopictus* (Skuse, 1894), and *Ae. terrens* Walker, 1856. Both genera are epidemiologically important as they are known vectors of arboviruses such as dengue and yellow fever.

The abundance of *Hg. leucocelaenus* (63%)*, Hg. janthinomys* (75%), and *Ae. terrens* (58%) was greater at the height of 3 m, while *Ae. albopictus* (52%) was more abundant at ground level (Table 1).

**Table 1:** Relative abundance, Shannon index and total number of culicids collected in Córrego da Luz, Casimiro de Abreu, Rio de Janeiro, Brazil, from July 2018 to December 2019.

Species	Ground level		3 m		Total
	N	(%)	N	(%)	N
*Hg. leucocelaenus*	113	37	192	63	305
*Hg. janthinomys*	1	25	3	75	4
*Ae. albopictus*	554	52	521	48	1075
*Ae. terrens*	27	42	38	58	65
Total	695	48	754	52	1449
Shannon's Diversity Index	0.6		0.8		
	0.7				

There was no statistically significant difference in the number of individuals of *Hg. leucocelaenus* (p = 0.3), *Hg. janthinomys* (p = 0.4), *Ae. albopictus* (p = 0.9), and *Ae. terrens* (p = 0.7) collected at the ground level and 3 m high. At the height of 3 m, a significant difference was observed (p ≤ 0.05) between the numbers of *Hg. leucocelaenus* and *Ae. terrens* (p = 0.03) and a very significant difference (p ≤ 0.01) between *Hg. leucocelaenus* and *Hg. janthinomys* (p = 0.003). Similarly, a very significant difference was also observed between *Hg. janthinomys* and *Ae. albopictus* (p = 0.001).

At ground level, there was a significant difference in the abundance of *Hg. leucocelaenus* and *Hg. janthinomys* (p = 0.02), and a very significant difference between *Hg. leucocelaenus* and *Ae. albopictus* (p = 0.002). There was also an extremely significant difference (p ≤ 0.001) between *Hg. janthinomys* and *Ae. albopictus* (p < 0.0001). As for diversity, the height of 3 m proved to be the preferred habitat for most species, with greater values in both the Shannon (0.8) and Simpson (0.46) indices. A majority of the Culicidae specimens (52%) was detected at this height (Tables 1, 2).

**Table 2:** Simpson's index of culicids collected in Córrego da Luz, Casimiro de Abreu, Rio de Janeiro, Brazil, from July 2018 to December 2019.

Species	Ground level			3 m		
	N	Ni/N = pi	(pi^2)	N	Ni/N = pi	(pi^2)
*Haemagogus leucocelaenus*	113	0.16	0.03	192	0.25	0.06
*Haemagogus janthinomys*	1	0.00	0.00	3	0.00	0.00
*Aedes albopictus*	554	0.80	0.64	521	0.69	0.48
*Aedes terrens*	27	0.04	0.00	38	0.05	0.00
Total	695	1.00	0.66	754	1.00	0.54
Simpson Index			0.34			0.46

N, number of mosquitoes; No, number of mosquitoes of each species; pi, proportion of individuals of a species.

**Figure 1:**
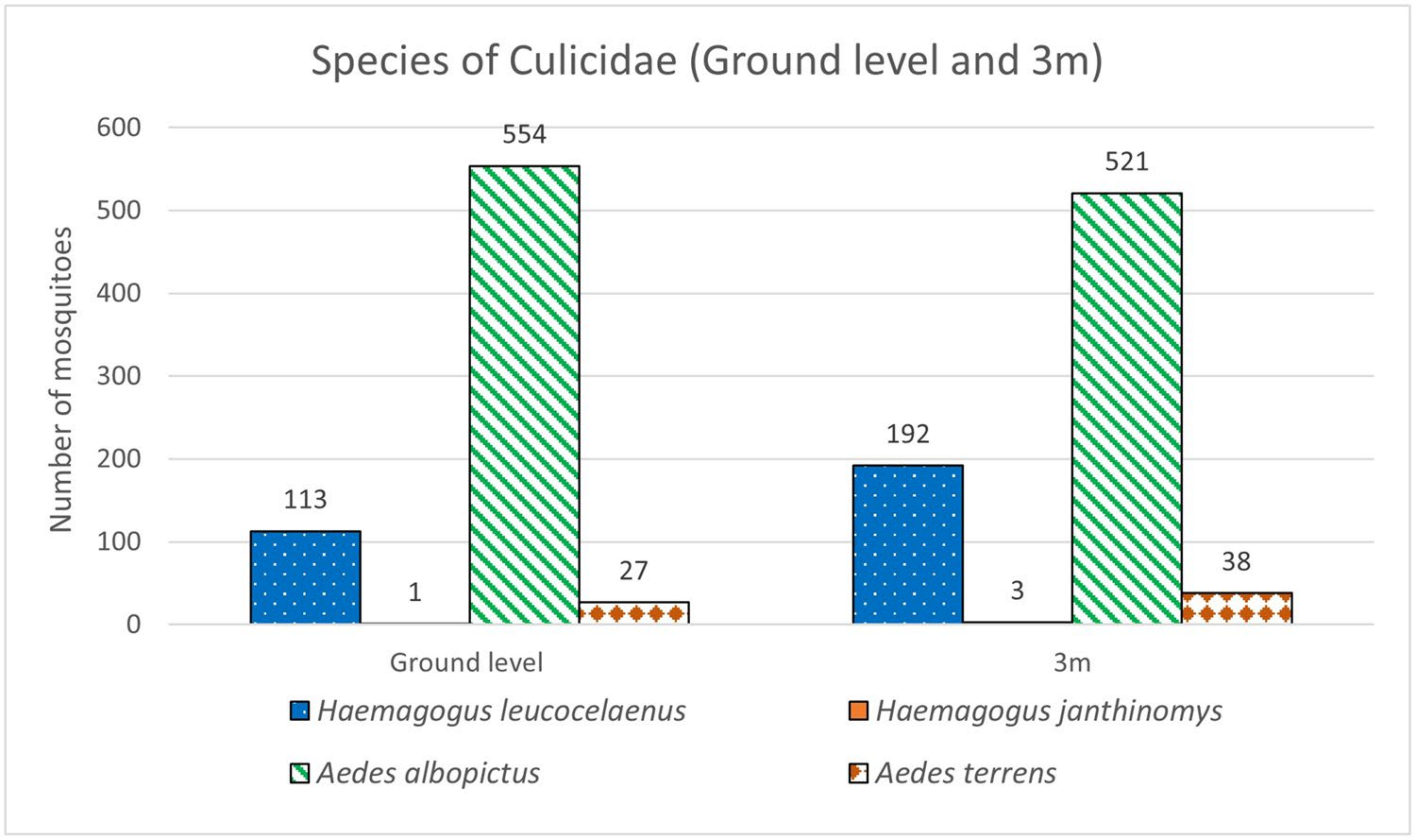
Species of Culicidae collected at ground level and 3 m, in Córrego da Luz Municipal Park, in the city of Casimiro de Abreu, Rio de Janeiro state, Brazil.

*Aedes albopictus* was the most abundant species, with 554 individuals collected at the ground level and 521 at 3 m high. This species was followed by *Hg. leucocelaenus*, with 113 individuals at the ground level and 192 at 3 m high. *Aedes terrens* and *Hg. janthinomys* were the least frequent species, with a total of 65 and four captured individuals, respectively (Fig. 1).

**Figure 2:**
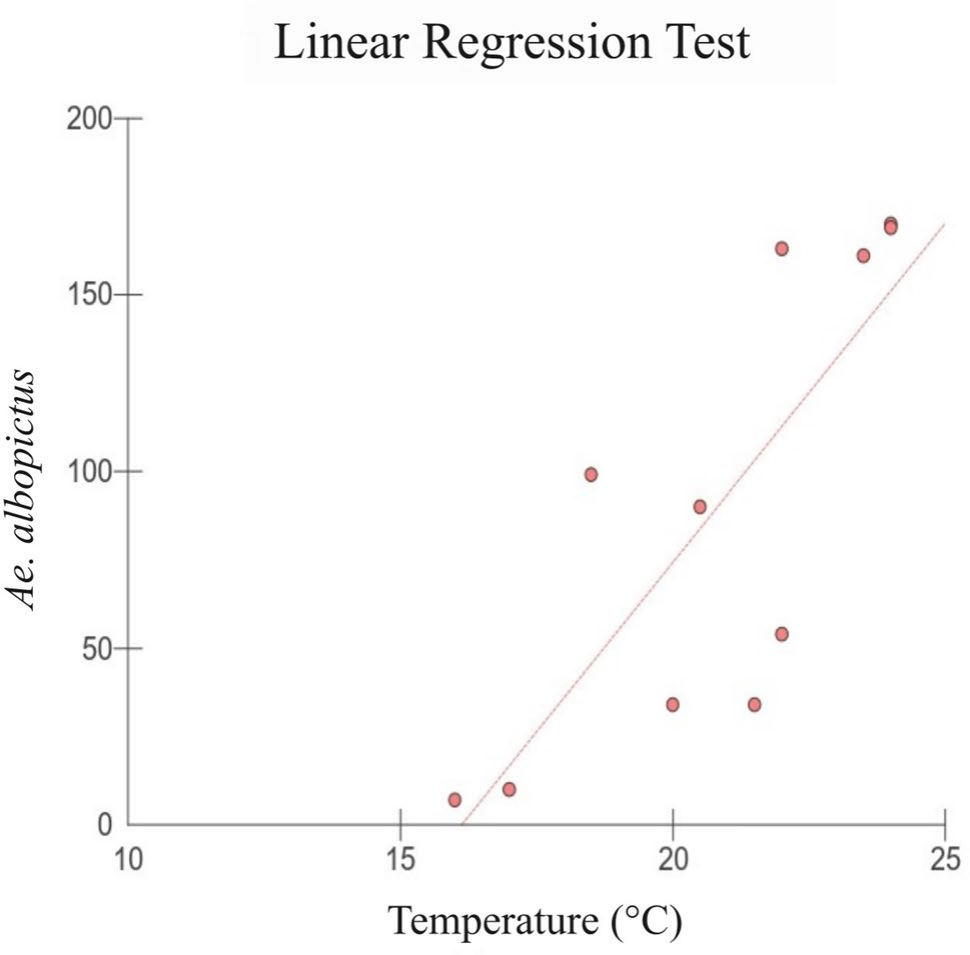
Linear regression test graph of *Ae. albopictus* related to temperature (ºC), in Córrego da Luz Municipal Park, in the city of Casimiro de Abreu, Rio de Janeiro state, Brazil.

The abundance of the two most common species, *Ae. albopictus* and *Hg. leucocelaenus*, were tested for correlation with rainfall and temperature (Brazilian Institute of Space Research – INPE, 2020) using linear regressions. Neither species was correlated with rainfall, though *Ae. albopictus* showed a significant positive correlation with temperature (p-value = 0.004; Fig. 2).

## Discussion

Studies of mosquito biodiversity in the Atlantic Forest contribute to a better understanding of the distribution patterns in mosquito populations. Specifically, possible changes in mosquito behavior and adaptations to the various environmental conditions or impacts in this biome are especially important^7^. Guimarães et al.^19^ observed that an increased incidence of epidemics related to anthropic environmental impacts is to be expected due to modifications in the behavior of pathogens and vectors.

Thus, the recent emergence of viral epidemics may be directly related to environmental impacts associated with human actions. This study was carried out in an altered landscape with fragments of the original Atlantic Rainforest ecosystem. *Aedes albopictus*, *Haemagogus leucocelaenus,* and *Hg. janthinomys* are very important epidemiologically due to their ability to transmit various arboviruses^8,20^.

In particular, the recent (2016–2019) outbreak of yellow fever in Brazil reinforces the importance of such studies in this biome^21^. Approximately 98% of the 2259 recorded human cases and 773 deaths occurred in localities in the Cerrado and Atlantic Forest biomes of southeastern Brazil^22^. The highest number of cases was reported in 2017, including one death in Casimiro de Abreu, the sampling area of this study.

Entomological studies conducted during the 2016–2019 outbreak in southeastern Brazil pointed to *Hg. leucocelaenus* as the primary vector of YFV along with *Hg. janthinomys,* while some species of *Aedes* and *Sabethes* were considered secondary vectors^23^. Cunha et al.^23^ observed that *Hg. leucocelaenus* was abundant in the foci of the disease and detected high rates of natural infections.

In addition to its role in the transmission of YFV, *Hg. leucocelaenus* has been experimentally shown to be able to transmit the chikungunya virus^24^. Furthermore, a fragment of the DENV-1 virus has already been found in some *Hg. leucocelaenus* specimens collected in northeastern Brazil^25^. *Aedes albopictus* is a competent vector of dengue and zika^26^, among other arboviruses^27^. This species can also be an intermediate vector for yellow fever between wild and urban areas^28^.

In Brazil, the research programs that led to studies on the vertical distribution of acrodendrophic mosquitoes were intended to clarify the transmission of wild yellow fever and simian malaria^29^. Studies on vertical stratification expand the ecological knowledge of the insect population in the forest, which is of fundamental importance for interpreting the biology of these critical species^30^.

Although there was no significant difference in the abundance of *Hg. leucocelaenus* and *Hg. janthinomys* at ground level and a height of 3 m, the overall abundance and diversity rates were higher in the samples collected at the height of 3 m. These results are consistent with those of Alencar et al.^9,10,31^, that populations of *Hg. leucocelaenus* in southeastern Brazil preferentially lays their eggs in the highest tree strata. Forattini^32^ suggested that *Haemagogus* spp. females search predominantly for blood meals at the highest levels of a treetop. It seems that these species can oviposit at a wide variety of heights in the Atlantic Forest^9,10,31^.

According to Consoli and Lourenço-de-Oliveira^33^, *Hg*. *janthinomys* also shows a clear preference for biting at the highest strata of the forest. Overall, we observed that specimens of *Hg. leucocelaenus* presented an eclectic, opportunistic, and adapted behavior regarding the search for hosts in the flight trend concerning the oviposition traps installed at different stratification levels.

On the other hand, individuals of *Ae. albopictus* showed a greater abundance and diversity at the ground level. Our results corroborate other studies showing that *Ae. albopictus* has an oviposition preference for low tree heights and ground level^9,10,34^. The occurrence of *Ae. albopictus* only in ovitraps at low levels can be attributed to the high availability of potential larval habitats. In this context, Obenauer et al.^35^ suggested that a potential decrease in habitat availability caused a more opportunistic and variable oviposition behavior. The presence of *Ae. albopictus* and other species susceptible to infection and flavivirus transmission may also pose a risk for spreading the zika virus. There is growing concern about the possibility of the zika virus establishing wild transmission cycles due to the high diversity of potential vectors, enhanced by the recent detection of this virus in neotropical primates^36^.

In this study, a significant difference was detected between the species found at the ground level and 3 m height. This finding may be related to the selection of sites at different heights for oviposition, which can be seen as a strategy to reduce interspecific competition^10^.

Weather conditions can also play a role in the vertical selection of oviposition sites^16^. Some studies have been shown that higher average temperatures favor mosquito eggs hatching, accelerates larval development^33,37^, and consequently speeds the YFV transmission^38^. Higher average temperatures also increase the frequency of biting activities^33^ and reduce the duration of YFV incubation in the vector, which is the time elapsed between the mosquito's infectious blood meal and the arrival of these viral particles in its salivary glands^39,40^. Thus, temperature plays an important role in the circulation of this virus. Therefore, monitoring the average temperature in risk areas can help to predict an increase in arbovirus transmission. Despite the unstable climate in southeastern Brazil, temperature and precipitation are critical abiotic factors in maintaining the YFV transmission throughout the year^41^.

In the present work, only *Ae. albopictus* was statistically positively influenced by temperature. This result corroborates the studies by Calado and Navarro-Silva^42^ and Docile et al.^43^, who found this species to be more frequent in breeding sites with high temperatures. However, the other vector species were not statistically significantly influenced by the climatic variables tested. *Haemagogus leucocelaenus* fluctuates in number of individuals throughout the year; however, the uninterrupted observation of eggs from this species in breeding sites illustrates that it maintains hematophagy activity throughout the studied months, regardless of climatic conditions^9,38^. This pattern of monthly egg collection and constant activity of *Hg. leucocelaenus* has also been described in other sites of the Atlantic Forest biome in southeastern Brazil^9,10^.

Monitoring the population dynamics of yellow fever vectors in areas of increased transmission risk is a critical component of the yellow fever health surveillance system. Elucidating the acrodendrophilic elements of these vectors’ oviposition and the effect of climatic variables that may influence these dynamics can be useful for yellow fever control programs by assisting in the development and improvement of health surveillance procedures by providing relevant biological data.

The results of our study provide information of interest for prophylaxis and control strategies, such as the definition of expanded risk areas and the prediction of silent virus circulation, which may help target the intensification of local vaccination campaigns in high-risk areas.

## Materials and methods

### Study area

**Figure 3:**
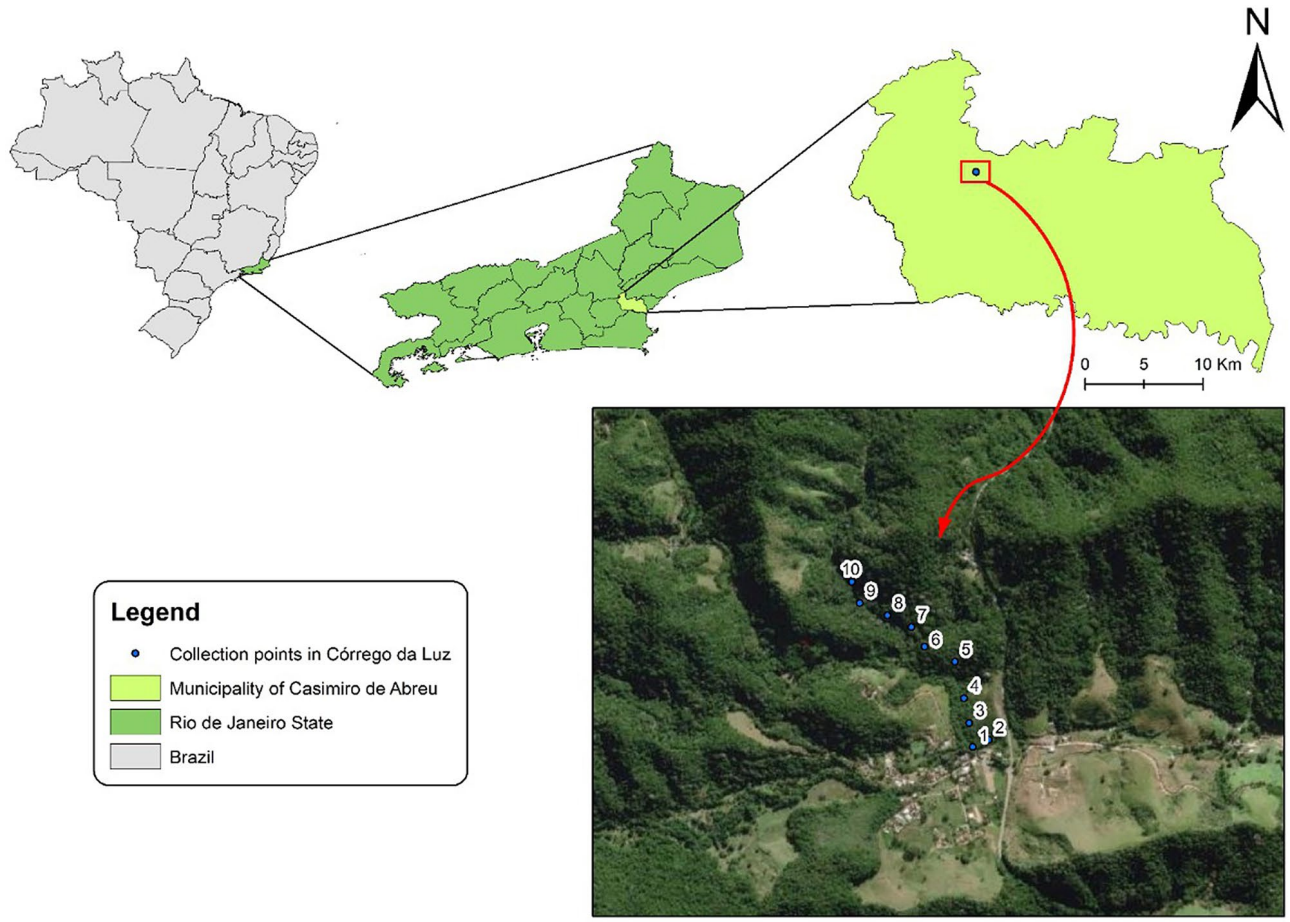
Sampling sites in the Córrego da Luz Municipal Park, located in the city of Casimiro de Abreu, Rio de Janeiro state, Brazil. Maps were prepared in ArcGIS Desktop: Release 10.5. Redlands, CA (ESRI) (https://www.esri.com/en-us/arcgis/products/arcgis-pro/overview/. Accessed: November 2020).

The Córrego da Luz Municipal Park is located in Casimiro de Abreu, Rio de Janeiro state, southeastern Brazil. The original vegetation cover of the region is typical of alluvial Atlantic Forest communities (herbaceous vegetation), dunes, and restingas (tree and herbaceous vegetation). Bergallo et al.^14^ reported that remnants of the Atlantic Forest are scarce. However, representative forest fragments do remain, as well as significant patches of secondary vegetation in advanced successional stages in the hills and coastal massifs, especially in the Rio de Janeiro municipalities of Maricá, Saquarema, and Silva Jardim (Fig. 3).

### Sampling

Ovitraps were used for collecting Culicidae eggs. These are composed of a black 500 mL pot with no lid. They also have three wooden paddles each (Eucatex plates), vertically held inside the trap by clips, an infusion of dry leaves and natural water is added into the ovitraps to reproduce a microecosystem as close as possible to the mosquitoes’ natural habitat^10,15^ (Alencar et al. 2016; Silva et al. 2018).

A total of twenty traps were installed at random intervals, with two traps per tree (one at ground level and another one at 3 m above the ground), between July 2018 and December 2019. The traps were installed at ten different sampling sites under the same height conditions, as follows: site 1, 22°28′32.9″ S 42°12′07.01″ W; site 2, 22°26′54.8″ S 42°12′23.3″ W; site 3, 22°26′49.1″ S 42°12′24.8″ W; site 4, 22°26′49.1″ S 42°12′24.8″ W; site 5, 22°26′46.0″ S 42°12′28.1″ W; site 6, 22°26′41.9″ S 42°12′30.8″ W; site 7, 22°26′38.1″ S 42°12′36.4″ W; site 8, 22°27′05.2″ S 42°13′53.7″ W; site 9, 22°26′51.2″ S 42°13′49.2″ W; site 10, 22°26′36.2″ S 42°12′37.6″ W.

The paddles in the traps were examined monthly, and taken to the Diptera Laboratory of the Oswaldo Cruz Institute, Rio de Janeiro. Positive paddles (containing eggs) were separated in the laboratory, had their eggs counted and were immersed in transparent trays containing dechlorinated water. The eggs were then placed in a controlled experimental environment with a thermoperiod regulated at a temperature of 28 °C ± 1 °C, relative air humidity of 75–90%, and a 12-h day/night cycle. These conditions allowed us to keep the specimens alive to adulthood for species determination.

In the laboratory, all the collected eggs were placed to hatch, with species determination upon reaching adulthood. After reaching the adult stage, the morphological characters were observed under a stereoscopic microscope, and the identification was carried out based on the dichotomous keys prepared by Forattini^16^ and Marcondes and Alencar^17^. The abbreviations of generic and subgeneric names follow Reinert’s^18^ proposal. After the species determination, all specimens were incorporated into the Entomological Collection of the Oswaldo Cruz Institute, Fiocruz, Rio de Janeiro, under the collection name “Atlantic Forest”.

### Statistical analyses

The number of individuals of each species found at ground level and 3 m was tested for normality using the Shapiro–Wilk test, followed by an analysis of independent samples, the Mann–Whitney test. The Shannon Diversity Index was used to evaluate the abundance of mosquitoes’ eggs collected at both ground level and 3 m high. The Simpson Dominance Index was used to measure the probability of two individuals randomly selected in the sample belonging to the same species; higher values of the latter index imply lower diversity levels. The relationship between the number of specimens per Culicidae species and the climatic variables of rainfall and temperature was shown to have a normal distribution through the Shapiro–Wilk test. The linear regression test was used to evaluate the relationship and influence of abiotic factors on the number of specimens per Culicidae species.

### Ethics statement

The permanent license for collecting and transporting zoological material from the Córrego da Luz Municipal Park within Brazil was granted by the Chico Mendes Institute for Biodiversity Conservation (ICMBio) through the Brazilian Biodiversity Information and Authorization System (SISBIO), under permit number 34911-1 dated 06/14/2020.
